# Editorial: Insights in social psychiatry and psychiatric rehabilitation: 2022

**DOI:** 10.3389/fpsyt.2024.1376580

**Published:** 2024-02-27

**Authors:** Andrew Molodynski

**Affiliations:** Department of Psychiatry, Medical Sciences Division, University of Oxford, Oxford, United Kingdom

**Keywords:** social psychiatry, mental health care, community care, co-production, rehabilitation

The articles in this Research Topic beautifully capture some of the most important themes in modern mental health care, and indeed in healthcare generally. Through what is proving to be a most difficult decade so far for humanity generally, with prolonged, devastating and traumatic conflicts around the world, the importance of connection, social capital, and partnership in healthcare has continued to gain ground. Health care providers and those who use services working together to find new ways of treating and managing conditions, new ways of measuring outcome, and indeed new ways of defining what ‘success’ or recovery looks like are very welcome indeed.

This Research Topic of very different articles has human experience and innovation as it’s common strand. The authors come at the problem from very different angles, and all give us useful insights in different areas and using a variety of methods. Chirio-Espitalier et al. explore the recovery construct in bipolar disorder as defined by personal rather than clinical or functional perspectives, concluding with numerous suggestions for further research and the refinement of practice. Lakshman et al. in Malaysia tested a Malay language version of a tool for assessing recovery orientated knowledge amongst health staff. They concluded that an adapted short version showed higher levels of validity and reliability- a real win as it will be easier to use in large scale studies and clinical practice. von Peter et al. reported on their co- produced instrument for measuring fulfilment of needs and experiences in psychiatric treatment (NEPT), in what was a striking example of career researchers and experts by experience working alongside each other. With many mental health scandals still occurring around the world, both in institutions and in the community, such collaborations could and should play a crucial part in ensuring that what is measured (and thus monitored) is appropriate and patient centred. Leading on from this, Umucu et al. reported on their fascinating and important work analysing so-called ‘character strengths’ in large groups of people with different disabilities. They received detailed responses from nearly 12,000 people across more than twelve countries and found the top five strengths were love of learning, honesty, appreciation of beauty and excellence, kindness, and fairness. What wonderful blocks to build on when helping people move forward in their lives and a real foundation for empowering and strengths-based services! Their more detailed findings should help those developing rehabilitation pathways for different conditions to focus on areas of strength that can be utilised and built upon, hopefully leading to better experiences of care and better outcomes in time.

Several of the other papers in the Research Topic remind us just how important our overall environment is and of the significant challenges that people with mental health problems can face. Wu et al. reported high rates of PTSD (over 13%) in a population affected by natural disaster, in this case a tornado. These kinds of damaging weather events are already becoming more common and more destructive, and no doubt rates of psychological trauma will rise in step with this, especially in parts of the world that are most affected. Reporting from China also, Chen et al. eloquently describe the twin effects of severe mental illness and homelessness and the inequalities that can lead to the latter, such as having lower levels of education and/or being from a minority group. Cheng and Lai examine another important issue with a systematic review of stress in the parents of children with special educational needs, finding that challenging behaviour, financial stress, and social isolation all contribute to negative feelings. Conversely good professional support and positive relationships are helpful and protective.

The final three papers in the Research Topic all look at possible ways forward. Asher et al. conducted detailed interviews with people taking psychotropic medication and describe complex interacting themes around feelings, beliefs, and emotions that are important for medication compliance and that are useful to understand when prescribing. Hug et al. conducted research among psychiatric inpatients, with many reporting that work and education were not addressed with enough priority by the treating team. 76% of those asked expressed a need for support, with 92% of those asking for job coaching. Finally, Tan et al. developed and piloted a gamified augmented reality approach to vocational skills in people with learning disabilities and found encouraging results and excellent tolerability.

The span of these Research Topic shows just how vibrant social psychiatry remains and how crucial it is to the human condition and to attempts to improve the quality of life of those affected by mental health problems.

## Author contributions

AM: Writing – original draft, Writing – review & editing.

